# Toward a molecular understanding of adaptive immunity: a chronology, part I

**DOI:** 10.3389/fimmu.2012.00369

**Published:** 2012-12-06

**Authors:** Kendall A. Smith

**Affiliations:** Division of Immunology, Department of Medicine, Weill Medical College, Cornell UniversityNew York, NY, USA

**Keywords:** clonal selection theory, blastogenic factor, lymphocyte activating factor, MHC restriction, antigen-specific factors, T cell growth factor, T cell (antigen) receptor, Ia molecules

## Abstract

The adaptive immune system has been the core of immunology for the past century, as immunologists have been primarily focused on understanding the basis for adaptive immunity for the better part of this time. Immunological thought has undergone an evolution with regard to our understanding as the complexity of the cells and the molecules of the system became elucidated. The original immunologists performed their experiments with whole animals (or humans), and for the most part they were focused on observing what happens when a foreign substance is introduced into the body. However, since Burnet formulated his clonal selection theory we have witnessed reductionist science focused first on cell populations, then individual cells and finally on molecules, in our quests to learn how the system works. This review is the first part of a chronology of our evolution toward a molecular understanding of adaptive immunity.

## INTRODUCTION: THE LEGACY OF BURNET

The term adaptive immunity is usually reserved for that type of immunity that adjusts in order to respond to an invading microbe, i.e., it adapts. A synonym that has also been used is “acquired” immunity, which is to say that it is different from “innate” or inborn immunity. Adaptive immunity carries with it the connotation of a heightened response to the re-exposure to an antigen experienced previously. This we call immunological memory. It is now recognized and well accepted that lymphocytes are the cells responsible for adaptive immunity, and that the two major types of lymphocytes, B cells and T cells, are both active participants. The phenomenon of immunological memory depends upon specificity of antigen recognition, as well as specificity of the immunological response. As such, adaptive immunity explains why vaccination is effective in preventing infectious diseases, and thus is the essence of immunity, defined as the *exemption* from disease.

When trying to understand any biological phenomenon it is often helpful to take a scholarly approach and delve into the past history of thought and experimental data that have been brought to bear on the problem. In this instance a logical starting point is the discussion of “The Facts of Immunity” as laid down by Sir Macfarlane Burnet in the third chapter of his seminal monograph of the Abraham Flexner Lectures that he gave at Vanderbilt University in 1958, entitled “The Clonal selection Theory of Acquired Immunity” (Burnet, 1959).

Burnet stated:

“*The facts of immunity that I want to summarize are those which seem most relevant to any attempt to look at the immune responses as a part of a general biological picture*.” They can be listed as follows:

1. The physical nature of the populations of reactive globulin molecules in a typical antiserum.
2. The differences, quantitative and qualitative, between primary and secondary responses.
3. The lack of immunological reactivity to body components and the related phenomenon of tolerance.
4. The qualitative types of immune response (i.e., cellular vs. humoral).
5. Congenital agammaglobulinemia.
6. The part played by mesenchymal cells, particularly those of the lymphoid series in immune reactions.

Of the nature of the reactive globulin molecules, considerable progress had already been made in the first half of the twentieth century by the time of Burnet’s lectures (Edelman, 1959; Porter, 1959). Now, 50 years later, we know that antibody activity is ascribable to immunoglobulin (Ig) molecules, which are identifiable in the sera of all vertebrates, and in mammals are comprised of five classes or isotypes, designated IgM, IgD, IgG, IgA, and IgE. Also, as a result of the progress made in the second half of the twentieth century, we know that Burnet’s theory of clonal selection is correct, each Ig molecule is the product of a single B cell, which differentiates into an Ig producing plasma cell (Fagraeus, 1948).

With regard to the differences between primary and secondary immune responses, as summarized by Burnet, “*A particularly clear example is that obtained with staphylococcal toxoid in early work* (Burnet, 1940)*, where the primary response is slow and of low titer, the secondary one rapid and rising almost logarithmically to a higher titer*.” We now know that the major difference accounting for the rapidity of the secondary response compared with the primary response, is owing to the proliferative expansion of the antigen-selected clones of cells during the primary response, as initially proposed by Burnet (Burnet, 1957, 1959). As to the qualitative differences between the primary and secondary responses, we also know that in the process of responding to the initial primary exposure to antigen the B cells undergo a differentiative process to become “memory” B cells, which has now been explained at the molecular level by genetic changes of recombination of the Ig genes (Hozumi and Tonegawa, 1976), thereby accounting for isotype switching, and somatic hypermutation that accounts for the phenomenon of affinity maturation (Bernard et al., 1978; Seidman et al., 1978).

Thus, for the past 30 years we have known *what* happens as a consequence of the primary antigenic stimulation, but we have only recently begun to unravel the secrets of exactly *how* these differentiative cellular changes take place at the molecular level, and what the molecular signals are that dictate them. Initially, it was assumed that antigen binding to surface Ig furnished all of the molecular signals necessary, in that after antigen selection, B cell proliferation ensues and precedes B cell differentiation. However, we are now aware that there are additional molecular ligand-receptor mechanisms that orchestrate these complicated cellular changes. It follows that it is axiomatic that B cell proliferation and differentiation are not simply pre-programmed changes that are only intrinsic to B cells and not other types of cells.

One crucial aspect of Burnet’s view of immunity that still had to be developed concerned the cellular immune response as compared with humoral immunity. By the time that Burnet formulated his theory, Medawar (1944) had shown that skin allografts prompt a remarkable rejection reaction with graft-infiltrative round inflammatory cells, and Chase (1945) had shown that it is possible to transfer cutaneous delayed-type hypersensitivity (DTH) to tuberculin with cells but not sera. Moreover, Bruton (1952) had reported a child with agammaglobulinemia who was unable to produce antibodies, and thus had great difficulty with bacterial infections, but had no difficulty recovering from viral infections.

Burnet first proposed that lymphocytes are the cells responsible for immunity (Burnet, 1957), and in his more extensive volume (Burnet, 1959), he summarized the available data indicating that there are at least three types of immune reactions:

1. Classical antibody responses
2. Hay-fever type responses
3. Tuberculin type responses

The first two types he was able to convincingly attribute to Ig molecules. However, the third type was problematic, in that “*Type (3) differs sharply in that there is no evidence that any circulating antibody is produced*” (Burnet, 1959). Burnet went on to discuss that perhaps lymphocytes might be responsible for these cellular reactions, but he was still unsure of the origin of lymphoid cells, and he speculated that perhaps all mesenchymal cells were interchangeable, including lymphocytes, monocytes/macrophages, plasma cells, and even fibroblasts. There seemed to be no controversy that plasma cells were the source of antibody molecules (Fagraeus, 1948), but there was a lack of convincing evidence of the interchangeability of each of these cells, especially as to whether lymphocytes could become plasma cells.

Because of the uncertainty of the cellular origins of immune responses, both humoral as well as cellular, Burnet could not furnish experimental support for his clonal selection theory. In his monograph, Burnet comes to the following conclusion:

“*Only by the use of a pure clone technique of tissue culture, which allows mesenchymal cells to retain full functional activity, would we be likely to find an answer. The clonal selection hypothesis would be completely validated if it could be shown that single cells from a nonimmune animal gave rise to clones, each cell of which under proper physiological conditions contained, or could liberate, antibody-type globulin of a single pattern*” (Burnet, 1959).

Of course, now with the advantage of hindsight, we know the answers to the questions posed by Burnet. However, it took another two decades to acquire the experimental data to prove the clonal selection theory, so as to make it the Clonal Selection *Law* of immunology. Moreover, Burnet was prescient in his prediction that only the capacity to develop pure clones of functional cells would make it possible.

## LYMPHOCYTES: THE CELLULAR BASIS FOR IMMUNITY

The initial breakthrough was supplied only 1 year later by Nowell (1960), who made the serendipitous discovery that a plant lectin extracted from the kidney bean, phytohemagglutinin (PHA), had the remarkable capacity to promote a morphological change in small round resting human lymphocytes to one in which the cells resembled immature leukemic blast cells, which became termed lymphocyte “blastic transformation.” Moreover, following this blastic transformation the cells underwent mitosis and cytokinesis. These findings were truly seminal, because prior to Nowell’s discovery, lymphocytes were described in textbooks as terminally differentiated, end-stage cells, incapable of self-renewal. Soon thereafter, Gowans et al. (1962) demonstrated that small lymphocytes would undergo proliferation *in vivo* after antigenic stimulation and give rise to circulating antibodies. Other reports followed soon thereafter that extended the phenomenon to specific antigen *in vitro* (Hirschhorn et al., 1963; Bach and Hirschhorn, 1964; Bain and Lowenstein, 1964). Accordingly, Burnet’s prophecy that antigen selected lymphocytes could undergo proliferative clonal expansion became a reality.

Also at this time, the capacity to visualize and enumerate antibody-forming cells (AFCs) *in vitro* was first reported by Neils Jerne together with Al Nordin and Claudia Henry (Jerne and Nordin, 1963). This technique, which came to be called the Jerne plaque assay, employed a source of lymphocytes from an animal immunized with sheep red blood cells (SRBCs), and a source of complement, which was supplied by using guinea pig sera. Thus, splenocytes or lymph node cells from SRBC-immunized rabbits or mice could be mixed with SRBCs and soft agar, and placed in Petri dishes, followed by the addition of complement, which would facilitate antibody-mediated lysis of the SRBCs, thereby forming a clear “plaque” against the homogeneous red background formed by the SRBCs. Each clear plaque could be observed under the microscope to contain a single central lymphoid cell, thus providing the first evidence that individual lymphocytes could give rise to cells that secrete antibody molecules. However, these data did not actually prove Burnet’s theory, in that the antibody molecules secreted by single cells still had to be shown to be “monoclonal” or identical individual Ig molecules.

In addition, the thymus had intrigued immunologists for some time, but experiments removing the thymus from animals failed to yield any immunological consequences, so that it was not clear whether this curious lymphoid organ played a role or not in the immune system. Since newborns were immunologically naive, Jacques Miller reasoned that the thymus might play a role in lymphocyte development, and thus he conjectured that “*neonatal thymectomy might be associated with some detectable effect on the maturation of immunological faculty*” (Miller, 1962).

Accordingly, Miller devised a method to thymectomize mice within the first 3 days of life (day 3 thymectomy; d3Tx). He found that such d3Tx mice grew normally during the first month, but thereafter suffered from “runting disease” very similar to graft vs. host disease (GvHD), “*characterized by progressive weight loss, lethargy, ruffled fur, humped posture and diarrhea*” (Miller, 1962). Most mice succumbed before 3 months of age with lymphocytes invading multiple organs. Within the first 6 weeks of life, when the mice looked grossly normal, the d3Tx mice were found to have a severe peripheral lymphopenia and undeveloped secondary lymphoid tissues. In addition, these mice were markedly immunocompromised, in that both skin allografts as well as xenografts went unrejected, and d3Tx mice failed to produce antibody agglutinin activity in response to common bacterial antigens. Accordingly, this was the first inkling that the thymus was important for the development of immunity, and Miller correctly concluded that “*during very early life, the thymus produces the progenitors of immunocompetent cells which mature and migrate to other sites*.” However, the cause of the runting disease that led to the premature demise of neonatally thymectomized mice went unexplored and unexplained.

In studies examining a secondary immune response of rabbits to the injection of a small molecular hapten coupled to a large protein carrier, Ovary and Benacerraf (1963) clearly showed that antibodies reactive with the hapten are increased only when the same carrier protein is used for the secondary stimulus as for the first injection. Also, if only the carrier protein is used for the second injection, an anamnestic response to the hapten does not occur. Eventually, this came to be called the “carrier effect,” although the basis for this phenomenon went unexplained.

Against this background the work of Cooper et al. (1965), 1966 in the chicken was seminal in defining two separate and distinct immune pathways. The avian Bursa of Fabricius, a lymphocyte-rich outpouching of the gastrointestinal tract much like the appendix, had been discovered serendipitously to be origin of precursors of AFCs (Glick et al., 1956). Thus, neonatal bursectomy led to the avian equivalent of Bruton’s agammaglobulinemia and the incapacity of producing antibody activity in response to immunization.

Accordingly, Cooper subjected newly hatched chickens to bursectomy, thymectomy or both, and then traced their development of secondary lymphoid organs, as well as their capacities to generate antibodies or to reject skin grafts. He found that like in mammals, “*thymus-dependent development is represented morphologically by the small lymphocytes in the circulation, and the white pulp-type development of the tissues.....and is basic to the ontogenesis of cellular immunity: graft vs. host responses, delayed hypersensitivity and homograft rejection.* By comparison, “*bursa-dependent development is represented by the larger lymphocytes of the germinal centers and the plasma cells, and functionally by the immunoglobulins*.” It was also noted by Cooper and his team that although neonatally thymectomized and irradiated chickens had normal circulating IgM and IgG levels, they only produced about half as much specific antibody responses to some, but not all antigens (Cooper et al., 1965). Such antigens were subsequently termed thymus-dependent or T-dependent antigens.

Thus, by 1965 adaptive antigen-specific immunity was established to derive from small lymphocytes that originated in the thymus, subsequently termed T cells, and in the bird in the bursa of Fabricius, subsequently termed B cells. A prolonged search for the mammalian equivalent of the avian bursa was negative, so that the default tissue became the bone marrow, assumed to be the source of antibody-forming mammalian B cells, and fortuitously also beginning with the letter B.

The year 1965 was also the year of the first reports of antigen-non-specific mitogenic activities produced by alloantigen-stimulated leukocytes. Two groups reported simultaneously that allogeneic mixed lymphocyte cultures produced “blastogenic factors” (Gordon and MaclLean, 1965; Kasakura and Lowenstein, 1965). Also, antiviral activity similar to interferon was found in mitogen-stimulated leukocyte culture supernatants (Wheelock, 1965). These findings were considered anomalous by most immunologists, because it was unclear how such antigen-non-specific activities, subsequently termed lymphokines, could participate in antigen-specific adaptive immunity. From this beginning, lymphokines were relegated to a secondary role, thought simply as amplifying antigen-initiated processes that were already ongoing. No one anticipated that lymphokines might be essential for adaptive immune responses to specific antigen, and crucial for immunoregulation.

## IMMUNE RESPONSE GENES AND CELLULAR COOPERATION

By far, some of the most exciting and intriguing findings of the 1960s were the reports by Baruj Benacerraf and his team of the genetic control of immune responses to synthetic polypeptide antigens in guinea pigs (Kantor et al., 1963; Levine et al., 1963; Green et al., 1966), and the confirmation and extension of these findings to inbred mouse strains by Hugh McDevitt and his group, who demonstrated that the genes in control were linked to those of the major histocompatibility complex (MHC; McDevitt and Tyan, 1968; McDevitt and Chinitz, 1969). However, exactly how this large and complex gene locus regulated the immune response remained totally obscure.

One clue was provided by Henry Claman’s group, who first showed that thymocytes and bone marrow derived cells cooperated in the production of antibodies (Claman et al., 1966). They took advantage of a system whereby potential immunocompetent cells are transferred to irradiated hosts, then immunized with SRBCs as antigen. They found that splenocytes were capable of producing foci of AFCs, but neither thymocytes nor bone marrow cells alone could do so. However, a combination of thymocytes + bone marrow cells were very active. “*The simplest interpretation is that one cell population contains cells capable of making antibody* (“*effector cells*”), *but only in the presence of* (“*auxiliary cells*”). However, their data did not allow them to discriminate the former vs. the latter. Conclusive experiments by Jacques Miller and George Mitchel using adult thymectomized, irradiated bone marrow-protected mice showed that “*AFC precursors were derived from bone marrow, while the role of thymus cells or of thoracic duct lymphocytes* (*TDL*)* is to influence in some way the differentiation of AFC precursors in response to antigen*” (Miller and Mitchell, 1969).

Subsequently, several technical findings advanced the capacity of immunologists to study the immune response. Up until the late 1960s the immune response, of necessity could only be studied *in vivo*, either in animals or humans. Mishell and Dutton (1967) made possible the analysis of the entire immune response *in vitro* by mixing naïve mouse splenocytes with SRBCs in culture for several days to promote a primary immune response, which they then quantified using a Jerne plaque assay. This facilitated examination of several aspects of the immune response that had heretofore been hidden in the proverbial *in vivo* “black box.” In particular it allowed the direct observation of Burnet’s prediction of the proliferative expansion of antigen-reactive clones (Dutton and Mishell, 1967). In addition, it also led to a dissection of the cells involved, and it was readily shown that macrophages are required for antibody formation to SRBCs by lymphocytes (Mosier, 1967). Also, in the words of Don Mosier, “*It appears that in the mouse spleen, production of antibody to sheep erythrocytes involves both antigen phagocytosis by macrophages and macrophage lymphocyte interaction, both processes being essential for development of lymphoid cells releasing hemolytic antibody.*”

Also, in a prescient report, Emil Unanue and Brigitte Askonas showed convincingly using radiolabeled protein antigen that “*immunogenicity of live macrophages persisted relatively unaltered for prolonged periods of time* (as long as two weeks) *and appeared to be associated with only a small percentage of antigen held by the cell in a form where it was protected from rapid breakdown and elimination*” (Unanue and Askonas, 1968).

Theodore Brunner’s team developed a radiolabeled ^51^Chromium-release assay that enabled the quantification of direct cell-mediated lysis (CML) of target cells, so that one could readily identify and quantify the amounts of lysis mediated by one cell population vs. another (Brunner et al., 1968). This assay extended the antigen-activation of lymphocyte proliferation to the capacity to monitor effector properties of cell-mediated antigen-specific adaptive immune reactivity *in vitro*, thus greatly facilitating the analysis of DTH, allograft rejection and GvHD.

Soon thereafter, using living cells rather than fixed or frozen cells, and using fluorescent-labeled antisera reactive with the theta (θ) antigen, which was known to be expressed preferentially by thymocytes and brain, Martin Raff identified θ-positive cells in a subset of murine peripheral secondary lymphoid tissues, and a reciprocal population of cells that were reactive with fluorescent-labeled anti-Ig (Raff, 1969; Raff et al., 1970). Pernis et al. (1970) confirmed and extended the expression of surface Ig on a subpopulation of rabbit lymphocytes, and speculated that this surface Ig could be the antigen receptors predicted by Burnet.

Martin Raff immediately tested the role of θ^+^ splenocytes (T cells) in a secondary humoral immune response in hapten-carrier primed mice (Raff, 1970). The experimental setup included adult mice immunized with the hapten 4-hydroxy-3-iodo-5-nitrophenyl acetic acid (NIP) coupled to the carrier bovine serum albumin (BSA). Splenocytes from these mice were harvested 8–12 weeks after immunization, treated or not with anti-θ and guinea pig sera as a source of complement, and then injected into syngeneic recipients previously irradiated with 600 rad. The following day the recipients were immunized with NIP-BSA, and then tested for circulating antibodies 10 days later. The results indicated that the T cells are “helper” cells, responding to the carrier determinants on BSA, whereas non-θ^+^ cells are the cells responsible for producing antibodies reactive with the hapten NIP. These findings were also confirmed and extended to the total *in vitro* immunization system by Schimpl and Wecker (1970).

Despite these new findings, the role of thymocytes and thymic-dependent cells in immunity was still obscure, leading Jerne just 1 year later to propose that “*in the primary lymphoid organs, e.g. in the thymus, the proliferation of lymphocytes....leads to the selection of mutant cells expressing v-genes that have been modified by spontaneous random mutation*.” (Jerne, 1971). He was partially correct about somatic mutation as responsible for the creation of antibody-antigen binding diversity, but totally wrong about thymocytes as precursors of AFCs.

## T CELL “HELP” AND HELPER FACTORS

Miller and Sprent (1971) were uniquely poised to perform adoptive transfer experiments to determine whether primed T cells and primed AFC precursors both possessed “memory,” and cooperated to produce a secondary immune response. To ensure complete depletion of thymus-derived cells these investigators employed a system of transferring TDL from antigen-primed mice to irradiated recipients. To test whether primed T cells were required, the thoracic duct cells were treated with anti-H2 sera that they had shown would eliminate virtually 100% of the T cells. Anti-θ sera were also used, but were found to only partially deplete T cells. These cells were then adoptively transferred to neonatally thymectomized mice: the antibody responses were lower by 1 log_10_ when T-depleted TDL were transferred. Moreover, primed B cells were also required in these adoptive transfer experiments, leading to the conclusion that “*memory is a property that can be linked to both B cells and T cells.*” (Miller and Sprent, 1971).

Given these findings, a series of experiments reported by Avrion Mitchison were seminal in a field that was to become obsessed with the question as to the molecular mechanism(s) of how T cells recognize antigens when they “help” antibody production, as well as the molecular mechanism(s) of the “help.” Mitchison’s experiments on the secondary immune responses of mice to immunization with hapten-carrier conjugates led him to conclude that the helper T cells recognize carrier determinants and the AFC precursors recognize either the hapten or other carrier determinants (Mitchison, 1971). He speculated that there was “*the possibility of an antigen bridge linking the receptor (presumably a normal Ig molecule) on the AFC precursor with another receptor (IgX) on the thymus derived cell*.” This concept came to be termed “linked recognition,” and led to a familiar schematic diagram of a B cell-antigen-T cell linkage presented at virtually all scientific meetings thereafter. Another term subsequently used for this function became known as “cognate recognition,” which emphasized the physical interaction between the AFC and the T cell, as well as the presumed molecular similarity between the B cell and T cell antigen recognition molecules.

Thus, the stage was set for the question that became the “holy grail” of immunology for the next decade, the nature of the T cell antigen receptor, which became abbreviated to simply the T cell receptor (TCR). Many of the most accomplished and prestigious research groups were attracted to the quest. Moreover, the focus of many immunologists also became the question as to how T cell “recognition” of antigen led to the apparent required T cell “help” provided to B cells that promoted antibody formation, as well as the molecular mechanism(s) responsible. The solutions to these questions consequently became intertwined.

Confounding these issues was the growing awareness that macrophages also seemed to play a role in the “help” that B cells required. It had also been shown that T cells needed “help” from macrophages to proliferate in response to stimulation by specific antigens and low concentrations of non-specific mitogens, such as PHA (Oppenheim et al., 1968). Accordingly, it was a logical next step to ask the question as to whether a soluble macrophage product might serve to replace the necessity of actual macrophages themselves. Bach et al. (1970) presented evidence that media conditioned by “*an adherent cell population-enriched in macrophages*” could substitute for macrophages allowing purified lymphocytes to proliferate in response to soluble protein antigens or to allogeneic lymphocytes. They speculated that the macrophages produced a factor that they termed “*conditioned medium reconstituting factor-CMRF*” that potentiated *in vitro* lymphocyte reactivity. The presumption was that the CMRF was produced by macrophages but worked its effects on the lymphocytes, amplifying their reaction to specific antigens.

Simultaneously, Richard Dutton and his group reported very similar results analyzing the generation of AFCs using their “Mishell–Dutton assay.” Thus, following up Mosier’s observation that macrophages are required for the generation of AFCs reactive with SRBCs *in vitro,* they found that conditioned media from 24 h cultures of glass-adherent cells, incubated with or without SRBCs, could substitute for macrophages in permitting the non-attached lymphocytes to generate AFCs (Dutton et al., 1970; Hoffman and Dutton, 1971). One other noteworthy aspect about the Mishell–Dutton *in vitro* AFC assay was its dependence on “optimal batches” of fetal calf sera (FCS) to observe any AFCs at all.

Soon thereafter, Igal Gerry working with Richard Gershon and Byron Waksman reported a series of experiments that showed that glass adherent cells released a mitogenic activity that enhanced the proliferation of murine thymocytes as well as purified peripheral T cells when stimulated by PHA (Gery and Waksman, 1972; Gery et al., 1972). They called this activity lymphocyte activating factor (LAF), and showed that its activity increased when the adherent cells are stimulated with bacterial lipopolysaccharide (LPS). As to its mechanism of action, it was speculated that LAF was simply a mitogen itself, like PHA. Alternatively, they wondered whether LAF supplied some trace nutrients necessary for cellular proliferation, thereby simply facilitating a proliferative process that was already initiated by the PHA. They referenced the earlier work on mitogenic activities found in leukocyte and macrophage conditioned media, but there was no way to ascertain whether the activities were identical or not.

Following up their findings of the requirement for T cells in the Mishell–Dutton assay, Schimpl and Wecker noted that allogeneic but not syngeneic thymocytes could substitute for θ^+^ splenocytes. They speculated that the strong alloantigenic stimulation of the thymocytes might have prompted them to produce a “potentiating factor,” similar to the leukocyte-derived blastogenic factor described previously (Gordon and MaclLean, 1965; Kasakura and Lowenstein, 1965), and the macrophage-derived activities described independently by Bach and by Dutton, except now they were specifically looking for activities derived from T cells, not macrophages or just leukocytes. Using the Mishell–Dutton assay system for the *in vitro* generation of AFCs, they related new evidence that there might actually be two activities produced by alloantigen-stimulated T cells; (1) a T cell expanding factor (TEF), similar to the blastogenic factor described previously, and that acted best if added early to the cultures, but also (2) a T cell replacing factor (TRF), which acted best if added late to the cultures (Schimpl and Wecker, 1972). They further speculated that the TRF would act on B cells, facilitating their differentiation to AFCs, and in the Mitchison linked-recognition model, the soluble TRF would only be at work over a very short range, like a neurotransmitter, and would only be capable of “helping” the B cell linked to a T cell via an antigen bridge between their respective antigen receptors. Thus, they covered all bases. Also, now it seemed pretty clear that for an antibody response to a T-dependent antigen, three distinct cells were required, B cells, T cells, and macrophages, and that soluble activities were produced by both macrophages and T cells that could replace the cells themselves.

## T CELL SUPPRESSION AND IDIOTYPIC “NETWORKS”

Two additional concepts were introduced at about this time that had tremendous influence on immunological thinking as well as experiments for the next decade. Richard Gershon reported that there were “suppressor T cells,” in addition to “helper T cells,” and that these cells might be responsible for immunological tolerance (Gershon and Kondo, 1970, 1971; Gershon et al., 1972). Moreover, these cells were antigen-specific and could be induced by antigenic stimulation, so that the outcome of antigenic stimulation, immunological activation or tolerance, depended upon a balance between T cell help vs. T cell suppression. Many of Gershon’s experiments were quite complex, involving thymectomized, lethally irradiated, bone marrow reconstituted mice that were subsequently immunized with large doses of SRBCs. Moreover, in many of his experiments, cells were adoptively transferred between serial recipients, which were also irradiated and reconstituted with various cell populations. Even though the experiments were complicated, his work gained credence because it was preformed *in vivo.* However, the mechanisms whereby these suppressor T cells functioned were obscured by the proverbial *in vivo* black box. Gershon speculated that perhaps they secreted a suppressive antigen-binding molecule, which he termed IgY to distinguish it from Mitchison’s putative secreted helper factor, IgX. The immunological community was very receptive to Gershon’s concepts, because they promised ways to manipulate the immune response therapeutically, either to enhance or suppress it. Therefore, there was something in it for everyone.

Neils Jerne’s “Idiotypic Network Hypothesis” is the other, very highly influential concept introduced in the 1970s (Jerne, 1974, 1976). To account for experimental observations that the antigen-binding, v-region of antibody molecules, which he termed a paratope, could itself serve as an antigen or a unique epitope that could be recognized by other B cells and give rise to antibodies reactive with them, the terminology of idiotopes and idiotypes was introduced. Jerne proposed that the vastly diverse population of antibody molecules in a normal individual represents a vast collection of antigens (idiotopes) by virtue of the unique amino acid sequences of each v-region. Thus, “*antibody molecules can be recognized as well as recognize*.” Jerne hypothesized that prior to the introduction of an external antigen, the concentration of each idiotope was low enough so that the system displayed an “eigen” behavior, a mathematical-physics term that is translated from the German as “self” behavior. Thus, Jerne proposed that resulting from paratope–idiotope interaction, the system achieves a dynamic steady state, as its elements interact between themselves. However, with the introduction of a foreign epitope, the B cells recognizing it would be stimulated to proliferate, thereby increasing the proportion of epitope-reactive cells, which would then differentiate into high epitope-reactive antibody producing plasma cells, flooding the system with their idiotopes. The net effect would be to markedly increase the concentration of the E-reactive idiotopes, which would exceed the activation threshold of many more reactive B cells, leading to the production of many anti-idiotopes. Ultimately, Jerne envisioned that the growing network stimulated by the original epitope would feed back to neutralize the response to the original foreign epitope.

## MHC RESTRICTION

One aspect of the immune system not considered by Jerne was the nature of the molecules that T cells used to recognize antigens. Like most others at the time, Jerne assumed that the TCR would also be found to be an Ig-like molecule, so that he envisioned T cells and B cells to be interchangeable from the standpoint of their antigen recognition molecules. Moreover, there was no place in the network hypothesis for Ir-genes as regulators of immune recognition. According to Jerne, “*Benacerraf and McDevitt regarded inescapable the conclusion that there exists a class of molecules encoded by Ir-genes, which are responsible for recognition of specificity at the T cell level, and that these molecules are not immunoglobulins*” (Jerne, 1974).

In attempts to understand Ir-gene function, and shed light on the nature of Ir gene products, David Katz and Baruj Benacerraf took a genetic approach to examine the question as to whether B cells and T cells needed to be histocompatible for the generation of AFCs in response to hapten (DNP)/carrier (keyhole limpet hemocyanin, KLH) conjugates (Katz et al., 1973). They used F1 hosts (A × B strains) as recipients of hapten-primed B cells from one parent (e.g., A strain) and carrier-primed T cells from the other parent (B strain), and found that only histocompatible (syngeneic) combinations would cooperate to generate AFCs. They then also performed similar experiments *in vitro* with similar results. They speculated that the B cells might express an MHC-encoded “acceptor” molecule that interacted with a similar MHC-derived molecule either secreted by, or expressed on the surface of the T cell.

This report was followed soon afterward by a similar report from Rosenthal and Shevach (1973) who studied the histocompatibility requirements of macrophages and T cells for specific antigen-induced T cell proliferation. Using guinea pig cells, they found that “*efficient interaction of macrophage associated antigen and immunospecific T lymphocytes as measured by antigen-induced lymphocyte proliferation, only occurs when the macrophages and T cells are histocompatible (syngeneic). It is likely that this interaction is mediated by histocompatibility antigens themselves, or by the products of genes closely linked to the MHC.*”

Accordingly, these reports established that Ir genes linked to the MHC complex somehow were very important for T cell antigen recognition. Whether it was necessary that MHC-encoded molecules were expressed by all of the cells for a productive interaction to occur remained to be determined. However, it was tantalizing to think that the MHC region actually coded for the elusive TCR. In this regard, it is important to understand that the genetic experiments done at the time indicated that the helper effects on the generation of AFCs and on antigen-specific T cell proliferation did not map to the traditional serologically-defined MHC-encoded molecules. Instead, they mapped between the serological determinants in what was termed the MLC-derived locus, that later became the I-region, or the Ir region, for immune response region.

In the face of these findings, the reports by Zinkernagel and Doherty (1974a,b), who studied the histocompatibility requirements for cytolytic T cells to recognize and kill lymphocyte choriomeningitis virus (LCMV)-infected cells, were particularly informative. These investigators found that cytolytic T lymphocytes (CTL) could only kill virus-infected target cells if they shared MHC loci, and that the MHC-encoded determinants mapped to the regions defined serologically. To explain their results, they proposed that T cells expressed two TCRs, one comprised of and interactive with self MHC-encoded determinants on the target cell, and another reactive with virus-specified determinants (non-self), vs. the one TCR hypothesis, in which only one TCR reacted with either a virus-modified MHC-encoded molecule or a combination of virus + MHC-encoded molecules, the so-called “altered self” hypothesis. In follow-up studies of MHC restriction of virus-specific cytotoxicity across the *H-2* barrier, Zinkernagel concluded:

“*The results are compatible with the idea that T cells are specific for “altered self” or “altered alloantigen”, i.e., a complex of cell surface marker and viral antigen. Alternatively, explained with a dual recognition model, T cells may possess two independently, clonally expressed receptors, a self-recognizer which is expressed for one of the syngeneic or tolerated allogeneic K or D “self” markers, and an immunologically specific receptor for viral antigen*.” (Zinkernagel, 1976).

## ANTIGEN-SPECIFIC HELPER AND SUPPRESSOR FACTORS

Given these findings against the background of the reports of both macrophage-derived and T cell-derived “factors” found in leukocyte conditioned media, investigators began searching for soluble factors, of both “helper” and “suppressor” varieties, and both antigen-specific as well as antigen-non-specific. Taussig and Monro (1974) were among the first investigators to identify an antigen-specific T cell-derived “helper” factor that appeared to be comprised in part by MHC-encoded molecules. They produced their factor as follows: “*T cells* (from mice) *primed in vivo by specific antigen, were incubated together with antigen in vitro for 6-8 hours. The cells were then removed by centrifugation and the supernatant, containing the T cell factor, *(was)* transferred together with bone marrow cells and antigen into lethally irradiated, syngeneic recipients*. *After 14 days the direct plaque-forming cell (PFC) response to the antigen in the spleens of the recipients was measured and compared to controls receiving B cells and antigen but no factor, or B cells with T cells and antigen.*” In attempts to characterize the helper factor, they found that the activity was removed with an immunoadsorbent column prepared with anti-H-2^d^ sera, but not anti-H-2^k^ or anti-Ig. Based on their findings, they suggested that “*the T cell factor is the soluble expression of the T cell receptor*.”

Also at this time, Marc Feldmann used combined *in vivo* and *in vitro* methods to generate supernatants for study (Feldmann and Basten, 1972; Feldmann, 1974). First, “activated T cells” were generated by lethally irradiating mice (800–900 rad), followed by injection intravenously (IV) with 10^8^ syngeneic thymocytes, and intraperitoneally (IP) with antigens emulsified in Freund’s complete adjuvant. Then 6–7 days later, splenocytes from these mice containing the “activated T cells” were cultured together with antigen in the upper chamber of double-chambered Marbrook–Diener flasks. The fluid in the lower compartment was harvested after 40–48 h and termed “T cell supernatant.” Feldmann described three different kinds of activities in these supernatants. There was an “antigen-specific helper activity” that enhanced the generation of AFCs. In the same supernatants there was both an “antigen-non-specific helper activity” as well as an “antigen-specific suppressor activity.” To make things even more complicated, it was found that macrophages could abrogate the suppressor activities. Moreover, both antigen-specific helper and suppressor activities were absorbed by Sepharose beads conjugated with anti-mouse Ig, anti-κ-chain or anti-μ-chain sera. Thus, in contrast to Taussig and Monro, Feldmann speculated that his T cell-derived factors, both helper and suppressor, contained an Ig molecule that he termed IgT. Moreover, it was claimed that suppression by “specific factor,” which he attributed to IgT-antigen complexes, depended upon direct interaction with lymphocytes.

Tomio Tada’s laboratory used a somewhat different system to examine the molecular mechanisms responsible for the suppression of anti-hapten specific antibody responses by T cells primed with high doses of carrier proteins (Takemori and Tada, 1975). Both thymocytes and splenocytes from carrier-primed mice were isolated and sonicated. Cellular debris was removed by centrifugation and the cell-free supernatants were tested for their effects on the generation of AFCs by their IV administration to naïve mice immunized concomitantly with hapten-carrier conjugate together with pertussis vaccine as adjuvant. The extracts were found to contain a suppressive factor that depressed the capacity of the immunized mice to generate IgG antibody responses to the hapten. The activity was antigen-specific for the carrier protein used to produce the factor. Like the activity described by Taussig and Monro, immunoadsorbent columns prepared with antisera raised against the K-end of the MHC complex of the donor strain, but not by anti-Ig removed the activity. Also, immunoadsorbents made with the carrier protein antigen also removed the suppressive activity.

Thus, all of these reports were focused on these antigen-specific factors because they hoped that these elusive factors could lead to the molecular nature of the T cell antigen receptor. However, because each laboratory used different methods to produce and assay for their factor activities, the field was chaotic.

## T CELL SUBSETS

Inbred mouse strains became a very important resource for investigating genetic contributions to immunological reactions. In particular, Lloyd Old’s group introduced the concept of the identification of subsets of T cells with their description of the *Ly* gene loci that specified discrete alloantigens expressed by functionally distinct T cells (Boyse et al., 1968, 1971). It is noteworthy that the use of congenic mouse strains to produce alloantisera created very unique reagents available only to a few investigators who had access to the special congenic mouse strains. Also, large numbers of mice were required to produce only small volumes of precious antisera that could be used only for critical experiments. Edward Boyse investigated congenic mouse strains that differed at only one genetic locus and raised antisera by immunizing across reciprocal congenic mouse strains. The *Ly-1* and* Ly-2* gene loci, on chromosomes 19 and 6, were found to specify alloantigens expressed exclusively and invariably on mouse T cells. Moreover, each locus was found to have alternative alleles that determined the T cell surface antigens Ly-1.1 and Ly-1.2, and Ly-2.1 and Ly-2.2, respectively. Thus, Pawel Kisielow, working with Boyse, first showed that subsets of peripheral T cells express distinct alleles of Ly surface determinants (Kisielow et al., 1975). Previous reports established that there appeared to be at least two functional T cell subsets, one that proliferated to a large extent when stimulated by alloantigens in an MLC, and another that possessed most of the cytolytic activity (Cohen and Howe, 1973). Kisielow found that splenocytes could be distinguished, in that approximately two-third were Ly-1+ and possessed most of the proliferative capacity, whereas approximately one-third were Ly-2+ and possessed most of the cytolytic capacity.

These findings were confirmed and extended by Harvey Cantor, also working with Boyse (Cantor and Boyse, 1975a,b), who showed that there were actually three subclasses of peripheral T cells, in that ~50% expressed all three Ly antigens, 1, 2, and 3. These cells were thought to function as precursors of the other two distinct subclasses. They also showed that during the MLC, the function of the Ly-1+ population to amplify the cytolytic function of the Ly-2/3+ population, depended on Ia+ stimulator cells. In a subsequent report the T cell subclasses were studied after immunization with SRBCs. Cells of the Ly-1+ subclass were found to provide “helper activity” for the generation of both primary and secondary AFCs. By comparison, cells of the Ly-23+ subclass were found to suppress the generation of AFCs. Because cytolytic cells could not be further separated or distinguished from suppressor cells, the T cell cytotoxic/suppressor (T_c/s_) terminology was introduced to immunology for the first time (Cantor et al., 1976).

Subsequently, McDevitt’s group produced evidence indicating that a new region within the I-region of the MHC locus, which they termed I-J, coded for surface determinants found only on suppressor T cells (Murphy et al., 1976). These findings depended on antisera produced by congenic mouse strains that differed only in very restricted areas within the I-region of the MHC locus. Simultaneously, Tada’s group using similar strains and antisera, reported data indicating that their carrier-specific suppressor factors contained determinants encoded by the I-J subregion (Tada et al., 1976). Thus, all of these data resulted in provocative evidence that genes within the MHC locus encoded molecules, some surface and some apparently secreted, that were very important for T cell regulation of immune responsiveness. Some of the encoded molecules were expressed by macrophages and B cells, while others were expressed by T cells. However, the molecular nature of these determinants and activities remained obscure.

Because each of the investigators used different methods to generate and test their various factor activities, the field was definitely complicated, especially as each investigator claimed different functional attributes for their factor activities. Moreover, there was a striking lack of attempts to purify the molecules responsible for the various activities, so that molecular characterizations beyond assaying for reactivity with various antisera were non-existent. The difficulties inherent in the biochemical techniques available for protein separation and purification were part of the reasons that biochemical characterizations of the various activities had not been performed. At the time, the only available techniques were molecular sieves that separated proteins based upon their molecular sizes or charges. Polyacrylamide gel separation had not yet been described, and high pressure liquid chromatography (HPLC) also was not yet available. However, another, just as important impediment was the lack of rapid quantitative assays available for the activities, with many investigators dependent only on *in vivo* assays, which made the determination of activities in many fractions from biochemical separative columns virtually impossible.

## LYMPHOCYTE-CONDITIONED MEDIA

The only *in vitro* assays available to identify the molecules responsible for the various activities that had been found in leukocyte-conditioned media (Ly-CM) monitored one of three functions; proliferation, antibody formation, and cell-mediated cytotoxicity. Thus, it is noteworthy that Plate (1976) described a T cell-derived soluble activity that could replace helper T cells in the generation of CTL. However, she could not differentiate the CTL helper activity from blastogenic factor, or the TRFs described by Schimpl and Wecker or Taussig and Monro. Thus, based upon the assays used, by 1976 there were descriptions of a myriad of soluble activities in Ly-CM.

At about the same time, several investigators used repetitive alloantigen stimulation to maintain antigen-reactive cells in culture for longer than just a few days. There were varied reports of success ranging from 3 to 4 weeks to several months. H. Robson McDonald working with Jean Charles Cerottini and Theodore Brunner first reported that it was possible to maintain murine CTL in culture using weekly or bi-weekly repetitive alloantigen stimulation for periods as long as 2 months (McDonald et al., 1974). Svedmyr (1975) reported similar findings using human MLCs. He was successful in maintaining cells for as long as four months by re-stimulating them bi-weekly. Subsequently, Zivi Ben-Sasson working with Ira Green showed that it was possible to use soluble protein antigen-pulsed adherent cell monolayers to repetitively stimulate guinea pig lymphocytes for periods of 2–5 weeks (Ben-Sasson et al., 1975). Dennert and De Rose (1976) used similar procedures and repetitive murine MLCs to continuously culture alloreactive cells for as long as nine months. Accordingly, in a period of just a few years, investigators operating under the dogma that specific antigen activated the proliferation of T cells, found that T cells could be repetitively alloantigen-stimulated and cultured for prolonged periods.

Against this background, a report by Doris Morgan working in Robert Gallo’s lab serendipitously found that conditioned media from PHA-stimulated human lymphocytes promoted the long-term culture of human bone marrow T cells for periods as long as 13 weeks (Morgan et al., 1976). These investigators had been searching for a growth factor that could facilitate the growth of human acute myeloid leukemia cells (AML), and had used PHA-stimulated Ly-CM, because it had previously been reported to be a source for granulocyte colony stimulating activity (G-CSA; Cline and Golde, 1974). Because they had used bone marrow as the cell source for their cultures, which was know to contain immature precursor T cell cells, it was not clear whether the cells grown were immature or mature T cells, in that they had not demonstrated any physiological mature T cell function (Morgan, 1988).

Initially, it looked as though the cultured cells might be Epstein–Barr virus (EBV)-transformed B cells. However, experiments using fresh rather than PHA-stimulated Ly-CM could not support long-term T cell growth, and further tests indicated that >95% of the cells formed erythrocyte rosettes (E-rosettes) with SRBCs, which was the only marker known at the time for human T cells. Moreover, tests for myeloid markers were negative. Because PHA was a known T cell mitogen, the most plausible hypothesis was that the PHA in the Ly-CM itself was responsible. However, the use of fresh media + PHA could not support long-term T cell growth. Accordingly, the default hypothesis was that a soluble mitogenic factor or activity secreted into the Ly-CM by the PHA-stimulated cells might be responsible. In this regard, one is reminded of Peter Nowell’s conjecture that perhaps PHA stimulation caused the cells to secrete a soluble factor that actually provided the growth signal for the cells (Nowell, 1960), as well as blastogenic factor (Gordon and MaclLean, 1965; Kasakura and Lowenstein, 1965).

Additional experiments were performed using peripheral blood mononuclear cells (PBMCs) as a source of the long-term T cell cultures, thereby excluding the possibility that the cells selected for growth in the Ly-CM were exclusively immature T cell precursors. For these studies, we collaborated with Ruscetti and co-workers, and established that activation of the cultured T cells with T cell mitogens resulted in the production of IFN activity, an accepted functional characteristic of mature T cells as compared with immature T cells (Ruscetti et al., 1977). Even so, because the long-term T cell cultures were initiated and maintained using PHA-stimulated Ly-CM, the role played by the PHA vs. any putative growth factor was unclear. Also, because PHA was known to activate polyclonal T cell proliferation, it was impossible to probe for any antigen-specific functions to the cultured cells.

## CYTOLYTIC T LYMPHOCYTE LINES

At the time, we had already established systems using murine splenocytes to generate CTL capable of lysing both allogeneic and syngeneic leukemia cells (Gillis and Smith, 1977a). First, we found that if we performed repetitive mixed tumor lymphocyte cultures (MTLC), we could enhance the generation of CTL as much as 100-fold. Then, with secondary but not primary allogeneic MTLC, it was possible to generate short-term T cell growth and differentiation to CTL capable of lysing both allogeneic as well as syngeneic leukemia cells. To explain our results, we speculated that the strong stimulation afforded by the histocompatibility antigens may have produced an allogeneic effect factor (AEF), similar to that found to enhance T–B cell interaction in the generation of AFCs (Amerding and Katz, 1974). We also postulated that by using allogeneic leukemia cells as stimulators, we might generate several different clones of cells, some reactive with alloantigens and some reactive with syngeneic tumor-specific antigens. This interpretation was compatible with the findings of Zinkernagel and Doherty, in that presumably the tumor-specific antigen could be expressed either as a part of the self MHC-encoded molecule, i.e., altered self, or it could be distinct from the self MHC, requiring two TCRs for recognition. However, the nature of the T cell antigen recognition structure(s) was still an enigma.

Given the findings of Morgan and co-workers, we speculated that it might be possible to create tumor antigen-specific long-term cytolytic T cell cultures by the use of Ly-CM. Because Concanavalin-A (Con-A) was known to be a better mitogen for murine cells than PHA, we prepared Con-A T cell supernatants (which later was referred to by some as CATSUP), and seeded CTL derived from allogeneic repetitive MTLCs using friend leukemia virus (FLV)-transformed leukemia cells, hoping that functional CTL could be maintained with the Ly-CM. This was considered a long shot, because the immunological dogma indicated that only specific antigen would be able to promote T cell proliferation. However, the very first experiments worked beautifully, and the first antigen-specific, long-term CTL lines (CTLL) were created (Gillis and Smith, 1977b).

At the time of this report, the CTLL had been in continuous culture for 22 weeks, and their allogeneic as well as syngeneic Tumor cell cytolytic activity had increased >10-fold. The cells required Ly-CM derived from Con-A-stimulated normal splenocytes, while fresh media + Con-A could not support long-term growth, thereby suggesting that the Ly-CM contained an obligatory growth factor. We speculated that one of the important issues that could now be approached was the molecular nature of the growth factor activity in the Ly-CM, using the long-term CTLL as target cells in a bioassay. In addition, we also speculated that these long-term CTLL might also be useful in adoptive therapy for leukemias, since they were cytolytic for virus non-producer leukemia cells, which were most analogous to human leukemia cells.

## THE T CELL GROWTH FACTOR BIOASSAY

Prior to the development of the CTLL, Torgny Fredrickson and I had developed a microassay for quantifying erythropoietin (EPO), using mouse fetal liver cells (FLC) as the target cells, which are comprised of a large proportion of EPO-responsive erythroid precursor cells (Fredrickson et al., 1977; Smith et al., 1977). This assay was based on the fact that erythroid precursor cells proliferate and differentiate into hemoglobin producing cells under the influence of EPO. Thus, we found that EPO exerted a concentration-dependent increase in tritiated thymidine (^3^H-TdR) and radiolabeled iron (^59^Fe) incorporation into erythroid precursor cells with a peak reaction detectable after only 24 h of culture in microwells. The principles of the assay were adapted from interferon bioassays, which routinely employed doubling dilution titrations, and comparison of the 50% effective concentrations (EC_50_) using probability analysis (probit analysis; Jordan, 1972). The rapidity of this assay, requiring only an overnight culture, together with the capacity to readily quantify the EPO concentrations was novel at the time, and allowed us to perform adsorption experiments with FLCs, which suggested that EPO interacted with cells by means of cell surface receptors.

Accordingly, once we had developed CTLL, it was natural to adapt the EPO microassay for the quantification of the growth factor in Ly-CM that was critical for the continuous CTLL proliferation. The quantitative microassay that we developed was a first for lymphokines activities, in that previous bioassays had only scored the presence or absence of a particular activity (Gillis et al., 1978b). We coined the term T cell growth factor (TCGF) to designate that this particular activity was distinctive in its capacity to mediate the long-term growth of the CTLL. The ability to quantify the TCGF activity allowed us to perform experiments on the biological characteristics of this activity for the first time. For example, we found that TCGF activity could be quantitatively depleted from Ly-CM by both mitogen-stimulated lymphocytes and the CTLL. Moreover, only T cell mitogens such as PHA and Con-A or alloantigens could elicit TCGF activity, B cell mitogens such as LPS being inactive in this regard. In addition, the removal of T cells from splenocyte or PBMC populations markedly reduced their capacity for production of TCGF activity. However, as we noted, this did not rule out the involvement of other cells, especially macrophages, in TCGF production. We noted that similar T cell factors, such as those reported to enhance the generation of lymphoid blast cells, AFCs, or CTLs could very well be similar to or identical with TCGF, but there was no way to distinguish between these various activities. Thus, we closed our report with, “*It is our hope that the bioassay described in this report will be of use in future experimentation to approach the isolation, characterization, and purification of TCGF and similar factors*” (Gillis et al., 1978b).

The new quantitative TCGF microassay was helpful immediately in regard to whether the TCGF activity also had T cell differentiative activity that facilitated the generation of CTL. In a series of experiments we established unequivocally that “*TCGF amplifies the generation of cytotoxic T cells. . ..Depletion of TCGF from* (allogeneic) *MTLC depressed both the number and cytolytic activity of cells generated, whereas addition of TCGF to MTLC enhanced both of these parameters*” (Baker et al., 1978). Just like the EPO effect on FLCs, which leads to both the proliferation of erythroid precursors as well as their differentiation to RBCs monitored by the uptake of ^59^Fe into hemoglobin molecules, TCGF promoted both the proliferation and differentiation of CTL progenitors.

The TCGF bioassay also allowed us to test Ly-CM derived from various species. It was found that the mouse CTLL cells could also be maintained in Ly-CM from rat and man, but human T cells could not respond to mouse TCGF. Thus, we could use the mouse CTLL bioassay to quantify the TCGF activity in human Ly-CM, so that we could optimize human TCGF production. This led us immediately to the creation of the first long-term antigen-specific human CTLLs (Gillis et al., 1978a). These hCTLL were very similar, if not identical to the murine CTLL, so that these experiments confirmed our murine studies and by extending the phenomenon to the human, they underscored the biological generality of the continuous culture of antigen-specific functional T cells. These data also prompted us to predict that “*Such cells will undoubtedly prove useful for studies on the mechanism of LMC, and for characterization of T cell antigen receptors and* (other)* T cell surface markers*.” We also speculated that human tumor-specific CTLL might conceivably be used for adoptive immunotherapy for cancer. In addition, it seemed reasonable to assume that it should be possible to generate functional helper and putative suppressor T cell lines, which “*may provide a new means for the study of both the mechanism and regulation of T cell mediated immunity*.”

## Ia MOLECULES

At about this time, Benacerraf (1978) published an “Opinion” article that galvanized and focused investigators perplexed by Ir genes, Ia antigens and the nature of T cell antigen recognition. First, he reiterated the characteristics of Ir genes in the I-regions of the MHC of mammals, and proposed presciently the hypothesis that perhaps the Ia molecules formed an immunogenic complex together with peptide epitopes capable of stimulating T cells. He summarized research indicating that T cells recognize epitopes comprised of short specific chains of three to four amino acids, whereas antibodies often recognized epitopes formed by the tertiary structures of native proteins. Also, he stressed the importance of macrophage processing and presentation for T-dependent immune responses, a phenomenon that had been recognized and studied for more than a decade (Unanue and Askonas, 1968). He intentionally stated that his theory did not define the nature of the T cell antigen receptors, nor the hypothesis that there could be more than one receptor. In addition, he was careful to state that his hypothesis was compatible with the Zinkernagel–Doherty phenomenon where the K- and D-region encoded molecules were postulated to interact with viral antigens in a manner similar to Ia molecule–peptide interaction.

He also specifically stated that his hypothesis was not concerned with antigen-specific helper and suppressor factors bearing I-region-controlled determinants. In addition, he stressed that according to his hypothesis, macrophage Ia molecules could bind macrophage-processed fragments of autologous proteins, as well as foreign proteins, and that specific unresponsiveness to autologous antigens could not depend upon the absence of Ia–self peptide interactions, but rather must depend on specific unresponsiveness at the T cell level by either an active or passive mechanism.

Thus, Benacerraf crystallized his 20 years of work on the genetic control of the T cell immune response. The Ir gene control specified the curious nature of the antigens recognized by T cells, which differed markedly from the nature of antigens recognized by B cells and antibodies. Even so, the nature of MHC-encoded molecules, the peptide–MHC interaction and the TCR still remained elusive, including the one receptor vs. two receptor hypotheses.

## CONCLUSION

The 20 years after Burnet proposed his clonal selection theory were taken up with the elucidation of the arm of the immune system that was totally obscure in his time, that is the cellular arm, which we now know as the T cell immune response. It’s discovery and comparisons and contrasts with the humoral or B cell immune response led to the uncovering of many surprising phenomena, including that T cells “helped” B cells proliferate and differentiate into AFCs, and also “suppressed” antibody formation. Moreover, T cells themselves could proliferate and differentiate into “effector” cells capable of actually killing target cells that they recognized as foreign. Even more mysterious and surprising was the fact that T cells “recognized” foreignness differently than did B cells and antibodies, and even more surprising, both the T cell and B cell immune responses depended upon the genetic constitution of the host, somehow linked to the large gene locus that encodes histocompatibility. In addition, not only were there two distinct kinds of lymphocytes, T cell subsets were recognized for the first time. All of these findings occurred as the science of immunology made the transition from studies of whole animals and humans to the study of cells in cultures. This transition led to the discovery of additional lymphocyte products that were not antibodies, which came to be known as lymphokines or cytokines. However, exactly what these newly discovered molecules were, and how they figured into the immune system remained obscure. Thus, by 1980 the burning questions of immunology focused on these issues, and how could one reduce the complexity further, as well as how could one reduce the science from cell populations to individual cells, to molecules.

## Conflict of Interest Statement

The author declares that the research was conducted in the absence of any commercial or financial relationships that could be construed as a potential conflict of interest.
